# Integrative Pathogenicity Assay and Operational Taxonomy-Based Detection of New Forma Specialis of *Fusarium oxysporum* Causing Datepalm Wilt

**DOI:** 10.3390/plants11192643

**Published:** 2022-10-08

**Authors:** Imran Ul Haq, Siddra Ijaz, Nabeeha Aslam Khan, Iqrar Ahmad Khan, Hayssam M. Ali, Ernesto A. Moya-Elizondo

**Affiliations:** 1 Department of Plant Pathology, University of Agriculture Faisalabad, Faisalabad 38040, Pakistan; 2 Centre of Agricultural Biochemistry and Biotechnology, University of Agriculture Faisalabad, Faisalabad 38040, Pakistan; 3 Institute of Horticultural Sciences, University of Agriculture Faisalabad, Faisalabad 38040, Pakistan; 4 Botany and Microbiology Department, College of Science, King Saud University, P.O. Box 2455, Riyadh 11451, Saudi Arabia; 5 Facultad de Agronomía, Universidad de Concepción, Chillán 3820572, Chile

**Keywords:** bootstrap method, effectors, phylograms, host pathogenicity, secreted in xylem, integrative omics

## Abstract

Pathogenicity-associated genes are highly host-specific and contribute to host-specific virulence. We tailored the traditional Koch’s postulates with integrative omics by hypothesizing that the effector genes associated with host-pathogenicity are determinant markers for virulence, and developed Integrative Pathogenicity (IP) postulates for authenticated pathogenicity testing in plants. To set the criteria, we experimented on datepalm (*Phoenix dactylifera*) for the vascular wilt pathogen and confirmed the pathogen based on secreted in xylem genes (effectors genes) using genomic and transcriptomic approaches, and found it a reliable solution when pathogenicity is in question. The genic regions ITS, TEF1-α, and RPBII of Fusarium isolates were examined by phylogenetic analysis to unveil the validated operational taxonomy at the species level. The hierarchical tree generated through phylogenetic analysis declared the fungal pathogen as *Fusarium oxysporum.* Moreover, the Fusarium isolates were investigated at the subspecies level by probing the IGS, TEF1-α, and Pgx4 genic regions to detect the forma specialis of F. oxysporum that causes wilt in datepalm. The phylogram revealed a new forma specialis in F. oxysporum that causes vascular wilt in datepalm.

## 1. Introduction

*Fusarium oxysporum* is a soil-borne fungus with a broad host range and a significant impact on the economy, ranked as the fifth most crucial phytopathogen [1,2]. The *F. oxysporum* species complex (FOSC) comprises non-pathogenic and pathogenic members. Several non-pathogenic members of FOSC generally are saprophytes, whereas some reside as endophytes [3]. However, pathogenic members of FOSC are delineated as forma specialis based on host pathogenicity [4,5]. Forma specialis are morphologically indistinguishable [6], and their characterization at the subspecies level commonly revolved around bioassays; however, molecular approaches have made the taxonomic branches more authenticated [7,8]. The virulence facet of a pathogen against its specific host may be accredited to a gene or a gene complex to produce the host-specific toxins [9]. 

*F. oxysporum* is an established cause of vascular wilt in various plant species, resulting in severe plant and yield losses. The vascular wilt is attributed to conductive tissue browning and wilting, ultimately leading to death. In date palm, vascular fusariosis causes necrosis, chlorosis, premature leaf drop, discoloration in the vascular system, brown strips on the rachis, and one-sided death of fronds, leading to wiling of the whole palm [10]. Fusarium causes a systemic infection that damages the palm; parasitism begins with the spore germ tube or any propagule entering the host through root lids, wounds, root hairs, or cracks caused by developing lateral roots [11]. Fusarium’s parasitic phase involves hydrolyzing enzymes penetrating the roots [12]. The mycelium forms microconidia in the xylem, germinating to form new hyphae, penetrating adjacent xylem cells, and increasing the infection rate [13]. Fusarium hyphae colonize the apoplast of the cell and cause cell alteration, resulting in expressing symptoms [12]. When host plants release adjacent parenchymal cells into the vessels in self-defense, the fungus spores and mycelium cause vascular occlusion. This vascular occlusion in infected plants ruptures the water column [14]. It causes the lower portion of tree branches to wilt, eventually killing the entire plant. When the plant dies and rots, new spores enter the soil and can survive as chlamydospores for eight years under unfavorable conditions [15]. Infected suckers are primarily responsible for disease transmission from one location to another (offshoots). Due to systemic infection and the unavailability of Fusarium-free suckers, confirming the pathogenicity of Fusarium wilt in date palm is a critical step in disease research (sometimes pathogens reside in plants asymptomatically). Pathogenicity-associated genes encode the effector proteins and categorize the diverse host range and specificity [16,17]. The effectors’ proteins secreted by the pathogen in the xylem are secreted in xylem proteins encoded by Secreted in Xylem (SiX) genes. These small cysteine-rich effector proteins significantly contribute to virulence [18]. The SiX genes are located on lineage-specific mobile pathogenicity chromosomes, and fourteen SiX genes encoding effectors have been documented so far [19]. Considering the specific nature of effectors encoded by pathogenicity-related genes, we hypothesized host pathogenicity genes as virulence determinant markers. This hypothesis provided the foundation for developing the Integrative Pathogenicity (IP) postulates. 

Polygalacturonases, either exopolygalacturonase or endopolygalacturonase, are the group of enzymes involved in cell wall degradation. Polygalacturonase genes, TEF1-α, and IGS, have been used to discriminate the species at the subspecies level and validate the possible existence of a new forma specialis in *F. oxysporum* [20,21]. Therefore, in this study, to delineate the *F. oxysporum* taxonomy to the subspecies level, we analyzed endopolygalacturonase genes (Pg1, Pg5), exopolygalacturonase genes (Pgx1, Pgx4), translation elongation factor 1-α (TEF-1α), and the intergenic spacer (IGS) region of rDNA in phylogenetic analysis to detect the forma specialis of *F. oxysporum* that causes wilt in datepalm. 

## 2. Materials and Methods

### 2.1. Molecular Characterization of Fungal Isolates

Molecular characterization of morphologically characterized fungal isolates sampled from wilted tissues was carried out by exploring the genetic loci, internal transcribed spacer (ITS) region, translation elongation factor 1-alpha (TEF1-α), and RNA polymerase II second largest subunit (RPBII). The fungal genomic DNA was isolated by treating the harvested mycelial mass, from freshly grown cultures, using the GeneJET Genomic DNA Purification Kit (Thermo Scientific, USA). The PCR analysis was performed using the gene-specific primers listed in Supplementary Table S1. The PCR products of required sizes were eluted (Gel Purification Kit, FavorPrep) and cloned in pTZ57R/T, a TA cloning vector for sequencing (Eurofins Genomics DNA sequencing services, USA). The sequences were trimmed (BioEdit version 7.2.6.1) to obtain high-quality (HQ) sequences. In the individual dataset of each locus, the HQ sequence was appended with available sequences of other Fusarium species retrieved from the NBCI database (Supplementary Table S2). Each dataset was aligned through a multiple sequence alignment program, MAFFT (Multiple Alignment using Fast Fourier Transform). The aligned datasets were concatenated through the Geneious software (ver. 4.8.5), and phylogenetic tree construction was made by the PAUP* V4.0 software to unravel the taxonomic hierarchy of fungal isolates.

### 2.2. Tailored Pathogenicity Test

#### 2.2.1. Identification of Pathogenicity Genes

The fungal isolate from the wilted tissues of datepalm characterized as F. oxysporum on a morphogenomics basis was subjected to genomic analysis to identify Secreted in Xylem (SiX) genes (pathogenicity-related genes/effector genes). The fungal total genomic DNA was extracted using the GeneJET Genomic DNA Purification Kit (Thermo Scientific, USA), following the manufacturer’s instructions. DNA purity was estimated at 260/280 nm by a UV-vis BioSpectrometer (Eppendorf). DNA concentration was calculated by measuring absorbance at a wavelength of 260 nm (OD260), and sample dilutions were made and stored at −20 °C. The polymerase chain reaction (PCR) analysis was carried out using specific primers of Secreted in Xylem (SiX) genes (Supplementary Table S1). The temperature profile was initial denaturation at 95 °C for 7 min; a loop of 40 cycles comprised of denaturation at 95 °C for 45 s, annealing temperature (Supplementary Table S1) for 50 s and extension at 72 °C for 45 s, and a final extension at 72 °C for 8 min. The purified PCR amplicons of required sizes were sent for sequencing to Eurofins Genomics DNA sequencing services, USA. The similarity search of identified SiX genes’ generated sequences was made by blasting them in the Blastn homology search tool, and sequences were submitted to GenBank. 

#### 2.2.2. Plant Inoculation, Fungal Re-Isolation, and Identification

The greenhouse-grown plants were inoculated with the characterized fungal isolate. Forty-eight symptomless date palms suckers were used in this trial. All collected suckers were potted and left untreated for three months for proper growth and establishment of roots. Suckers were inoculated by making a shallow slit 2 cm lengthwise on the adaxial surface and a spore suspension was injected at 10^7^/mL of 20 mL volume separately using a hypodermic needle or syringe. Fourteen suckers were inoculated with the suspension, two control plants were treated with sterilized water only, and the treatment was replicated thrice. The inoculated area was wrapped with parafilm to prevent moisture loss. Suckers were covered with plastic bags for 24 h and kept in a greenhouse until symptoms appeared. The fungal re-isolation was made in inoculated plants from diseased tissues upon appearance of symptoms. The re-isolated fungi were subjected to molecular characterization based on the effector gene (SiX genes) associated with host pathogenicity by adopting the above procedure. 

#### 2.2.3. Expression Profiling While Bipartite Interaction between Host and Pathogen

The plant transcriptome was analyzed during pathogenesis for determining effectors’ genes expression. Total RNA isolation of infected leave samples was performed using the GeneJET Plant RNA Purification kit and RapidOut DNA Removal kit (Thermo Scientific, USA), and a quantified NANODROP (8000 Spectrophotometer, Thermo Scientific). The cDNA of each sample was synthesized with a RevertAid First Strand cDNA Synthesis Kit (Thermo Scientific, USA), dilutions (10 folds) were made and stored at −20 °C. Reverse transcriptase PCR (rtPCR) analysis using specific primers for Secreted in Xylem (SiX) genes was performed to determine the presence of host pathogenicity-associated effectors encoding proteins in the plant transcriptome under disease conditions. Then, real-time quantitative PCR analysis (CFX96 Touch Real-Time PCR detection system) was performed for the expression profiling of identified SiX genes upon infection under bipartite interaction between host and pathogen. The transcript levels of the *SiX1, SiX03, SiX06, SiX7,* and *SiX10* genes (target genes) were determined relative to β tubulin (reference gene). 

#### 2.2.4. Random Mutagenesis and Plant Inoculation with Mutated Pathogen

The characterized pathogen was mutated with different doses of a chemical mutagen, ethyl methanesulfonate (EMS). The spores of fungal isolates were treated with 0, 100, 500, 1000, 1500, 2000, and 2500 μg mL^−1^ EMS solution at 26 °C for exposure times of 24, 48, and 72 h. The treated spores were washed with sterile distilled water and were spread on a culture medium (PDA medium) at 26 °C. The cultures from viable treated spores with altered phenotypes were further characterized at the genomic level using a Secreted in Xylem (SiX) genes specific primer associated with host pathogenicity (as described above). The experiment was conducted in triplicate. The mutated versions of a characterized pathogen that have shown no PCR amplification were used to inoculate the plants to assess pathogen validity.

### 2.3. Pathogen Characterization for Unraveling Taxonomy 

#### 2.3.1. PCR Amplification

The endopolygalacturonase genes (Pg1, Pg5), exopolygalacturonase genes (Pgx1, Pgx4), translation elongation factor 1-α (TEF1-α), and intergenic spacer (IGS) region of rDNA were amplified in a polymerase chain reaction (PCR) using the gene-specific primer pairs given in Supplementary Table S1. The PCR analysis was conducted in a thermal cycler (VeritiTM 96 wells, Applied Biosystems) with a reaction volume (25 μL each) comprised of 15 ng of fungal gDNA; a primer pair (10 mM each), 12.5 μL Phusion High-Fidelity PCR Master Mix with HF Buffer, following a thermal program; 95 °C for 3 min, 35 cycles with denaturation at 95 °C for 50 s; annealing temperature (Supplementary Table S1) for 35 s, extension at 72 °C for 90 s, and final extension at 72 °C for 8 min. No template control (NTC) was run in all reactions as a negative control. PCR products were electrophoresed on high-resolution agarose gel (0.6%); required amplicons were eluted from the gel using a FavorPrep Gel purification kit (Favorgen Biotech Corporation, Taiwan), and sent to Eurofins Genomics DNA sequencing services, USA. All generated sequences were trimmed (BioEdit software) and subjected to the Blastn tool for searching their homology before depositing in the GenBank to obtain accession numbers (Supplementary Table S3).

#### 2.3.2. Phylogenetic Analysis

The individual dataset of each gene was made by supplementing the generated sequences with available sequences of other *F. oxysporum* forma specialis retrieved from the NBCI database (Supplementary Table S3). The individual dataset was aligned through the ClustalW program. The neighbor-joining (NJ) method for phylogenetic tree construction was implemented on each aligned individual dataset with MEGA6.06 (Molecular Evolutionary Genetics Analysis version 6.06) software by employing a phylogeny test and the Bootstrap method with 1000 bootstrap replications.

## 3. Results and Discussion

***Integrative Pathogenicity (IP) test*:** The virulence facet of a pathogen against its specific host may be accredited to a gene or a gene complex to produce the host-specific toxins [9]. We hypothesized the effector gene(s) associated with host-pathogenicity as determinant markers for virulence to develop Integrative Pathogenicity (IP) postulates. Keeping in view the specificity in nature and contribution to host-specific virulence, Secreted in Xylem (SiX) genes were used in the study; moreover, to set the criteria of integrative pathogenicity, we decided to focus on host plants where pathogenicity remains in question due to the presence of systemic microbes. Hence, we made trials on date palm, which is a highly regarded fruit crop around the globe, also known as the “tree of life” due to its nutritional profile, medicinal significance, excellent yields, long life, and high adaptability to diverse climatic conditions, soils, and geographic areas [22]. Fusarium wilt (vascular fusariosis) is a significantly destructive fungal disease of the date palm [10] caused by *F. oxysporum*. In this study, traditional Koch’s postulates were tailored to the modern field of biology. The tailored pathogenicity test was comprised of several procedural steps. In the first step, the fungal isolates from the wilted tissue of datepalm were cultured, purified, and characterized based on Secreted in Xylem (SiX) genes (pathogenicity related genes/effector genes) through PCR analysis. Out of 14 SiX genes-based primer pairs, only four primers gave amplification, *SiX1* (~250 bp), *SiX3* (~600 bp), *SiX6* (~700 bp), *SiX7* (~700 bp), and *SiX10* (~650 bp). Bateson et al. [23] documented that the deletion of *SiX1, SiX3*, and *SiX6* leads to reduced virulence in *F. oxysporum*; moreover, these genes were key role players in host-specific pathogenicity [18]. The purified PCR amplicons of required sizes were sent for sequencing to Eurofins Genomics DNA sequencing services, USA. The similarity search of identified SiX genes’ generated sequences was made by blasting them in the Blastn homology search tool, and sequences were submitted to GenBank. The sequences of SiX genes were assigned GenBank accession numbers, *SiX1* (MZ736624), *SiX3* (OK490370), *SiX6* (OK512877), *SiX7* (MZ714597), and *SiX10* (MZ803209). 

The greenhouse-grown plants were inoculated with the characterized fungal isolate. All challenged plants had shown typical wilt symptoms after forty days of inoculation (40 dpi); control plants (non-inoculated) showed no characteristic symptoms. The fungal re-isolation was made in inoculated plants from diseased tissues upon symptoms appearance. The re-isolated fungus from inoculated diseased plants was identified by molecular characterization based on effector genes (SiX genes) associated with host pathogenicity by adopting the abovementioned procedure.

**Figure 1:**
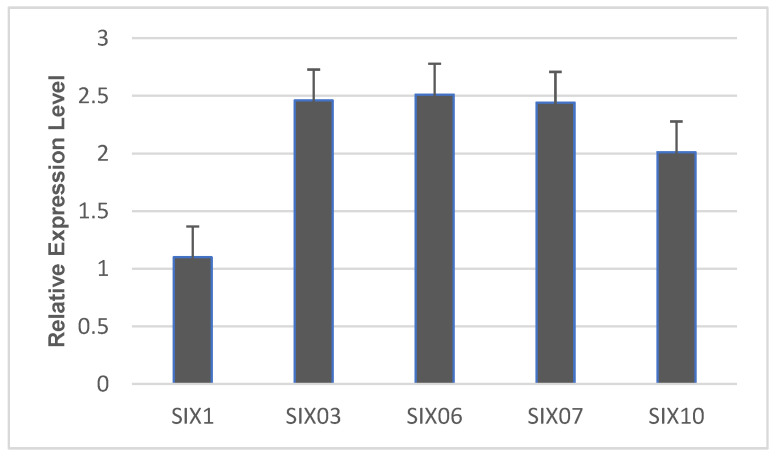
Expression profiling of Secreted in Xylem (SiX) genes in datepalm under *F. oxysporum* stress.

The transcriptomic analysis using reverse transcription polymerase chain reaction (rtPCR) showed the presence of effectors genes’ (SiX genes) transcripts in the transcriptome of diseased plants by amplifying the identified SiX genes. Upon infection, the expression profiling of SiX genes revealed *SiX3, SiX6*, and *SiX7* with significantly upregulated expression, followed by SiX10. However, the expression of *SiX1* was shown to be comparatively lower than other *SiX* genes (Figure 1). Duan et al. [24] also found upregulated expression of *SiX6, SiX7*, and *SiX10* during *F. oxysporum* infection in plants. Anabestani et al. [25] also identified and detected the putative effector genes of pathogens in the transcriptome of witches’ broom disease of lime (WBDL) infected plants.

The pathogen cultures of spores treated with 1500 μg mL^−^^1^, 2000 μg mL^−1^, and 2500 μg mL^−1^ EMS for 48 and 72 h (incubation time) showed phenotypic alteration on morphological testing. Mycelial mass was collected from cultures with altered morphology and subjected to a DNA isolation procedure. The PCR analysis using identified SiX gene-based primer pairs showed no amplification in the fungal cultures treated with 2000 μg mL^−1^ EMS in biological and technical triplicate repeats. The mutated fungal culture (2000 μg mL^−1^ for 48 h) inoculated the greenhouse-grown date palm plants, and control plants were inoculated with a typical pathogen. The plants inoculated with mutated culture showed no disease symptoms; however, control plants showed distinct typical wilt symptoms. These results validate the identified wilt pathogen in date palm as *F. oxysporum*.

We tailored the pathogenicity test by hypothesizing criteria and experimented on datepalm (*Phoenix dactylifera*) for vascular wilt pathogen (*F. oxysporum*). Moreover, we attempted to set the criteria on datepalm and then tested these postulates on other plants to evaluate their validity and efficiency, with successful results, and found it a reliable solution, even where pathogenicity is in question. Based on the results of tailored pathogenicity tests, we have given the Integrative Pathogenicity (IP) postulate in this study for efficient and authenticated pathogenicity testing in plants. This research study was carried out under PARB project no. 802 and the umbrella collaboration of the Fungal Molecular Biology Lab, Department of Plant Pathology, University of Agriculture Faisalabad, and the “Molecular Biology of Plant Disease Resistance Lab” of CABB, University of Agriculture, Faisalabad, Pakistan.

### 3.1. Integrative Pathogenicity (IP) Postulates

(1) The microbe must be present in all organisms affected by the disease in question.
(2) The isolated microbe must be characterized for the pathogenicity-related gene(s) (effector genes).
(3) The microbe inoculated in the host plant must be re-isolated upon disease appearance.
(4) The re-isolated microbe must be characterized for the presence of effector gene(s) associated with host pathogenicity.
(5) The plant transcriptome while pathogenesis due to bipartite interaction between host and pathogen must express the product(s) of effector gene(s).
(6) A host plant must be devoid of disease upon inoculation with a mutant version of the characterized pathogen.

**Figure 2:**
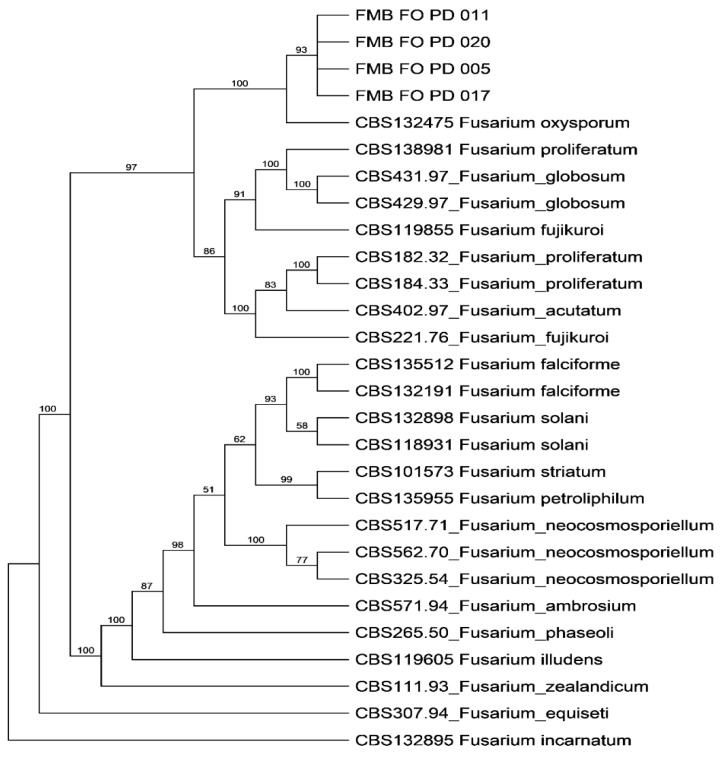
Phylogenetic tree of the concatenated dataset of the internal transcribed spacer (ITS) region, translation elongation factor 1-alpha (TEF1-α) and RNA polymerase II second largest subunit (RPBII) using PAUP* V4.0 software.

***Morphogenomic characterization of pathogen:*** The fungal culture exhibited white to creamy floccose aerial mycelium on PDA medium and was pale-violet from the inverse side of colony growth. Conidia (microconidia, microconidia) were thin-walled hyaline. Microconidia were 3.9–13.6 µm in length and 1.6–4.0 µm in width with elliptical, obovoid, oval, or reniform shape and had no or a single septation. Macro conidia were 31.6–45.3 × 2.8–5.4 in size, fusiform with tapered ends; apical cells were straight to slightly curved; most were narrower with 3–7 septations. These were the morphological attributes of *F. oxysporum*. Therefore, the phylogenetic analysis was performed to unravel the operational taxonomy of the proven pathogen causing vascular wilt in datepalm. The genetic regions ITS, TEF1-α, and RPBII of sampled fungal isolates were amplified through PCR analysis, and PCR products were directly sequenced. The PCR analysis amplified the DNA fragments of ~684 bp (ITS), ~710 bp (TEF1-α), and ~970 bp (RPBII). The sequences deposited in GenBank were assigned accession numbers (Supplementary Table S2). The phylogenetic trees constructed using PAUP* V4.0 software under heuristic search using the Bootstrap method with 1000 bootstrap replications revealed our fungal isolates in a well-separated clade close to the F. oxysporum strain CBS132475 with 93% bootstrap support. However, the phylogram (bootstrap consensus tree) showed that these isolates, including *F. oxysporum* strain CBS132475, were separated with 100% bootstrap support from other Fusarium species in a hierarchical tree (Figure 2). 

**Figure 3:**
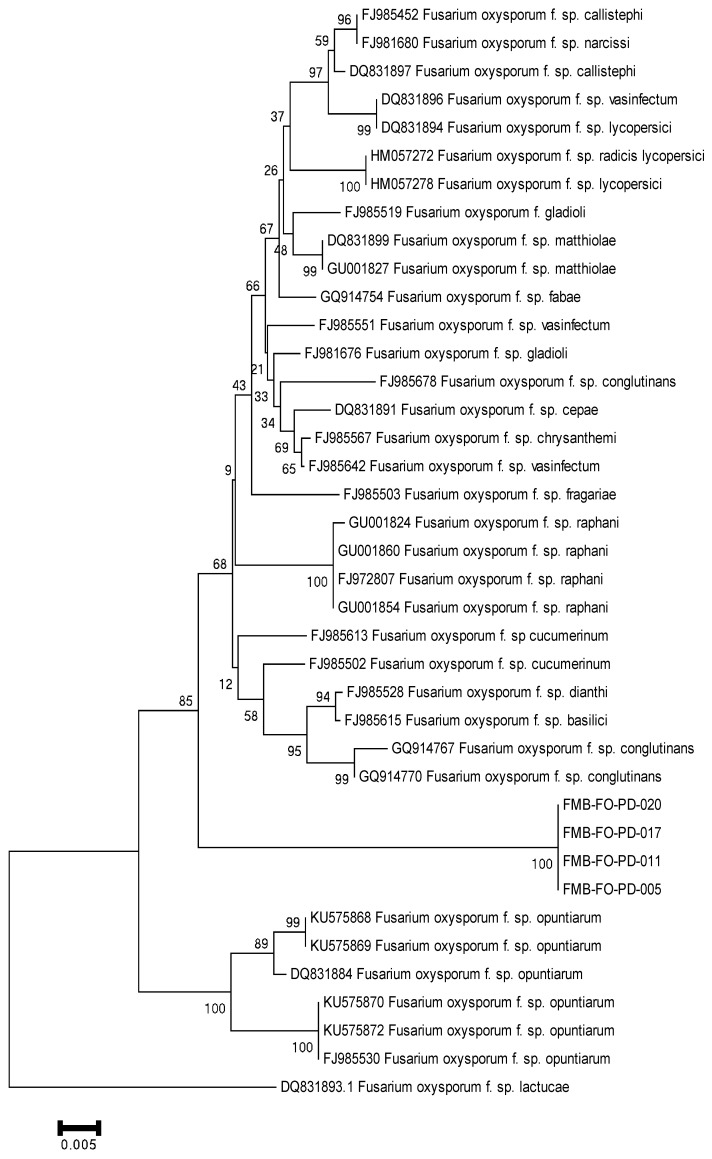
Phylogenetic tree based on the intergenic spacer (IGS) region of rDNA, using MEGA6.06 software under Neighbor-Joining (NJ) analysis, displayed a distinct and separate clade of the *F. oxysporum* isolates from other forma specialis with 100% bootstrap support.

**Figure 4:**
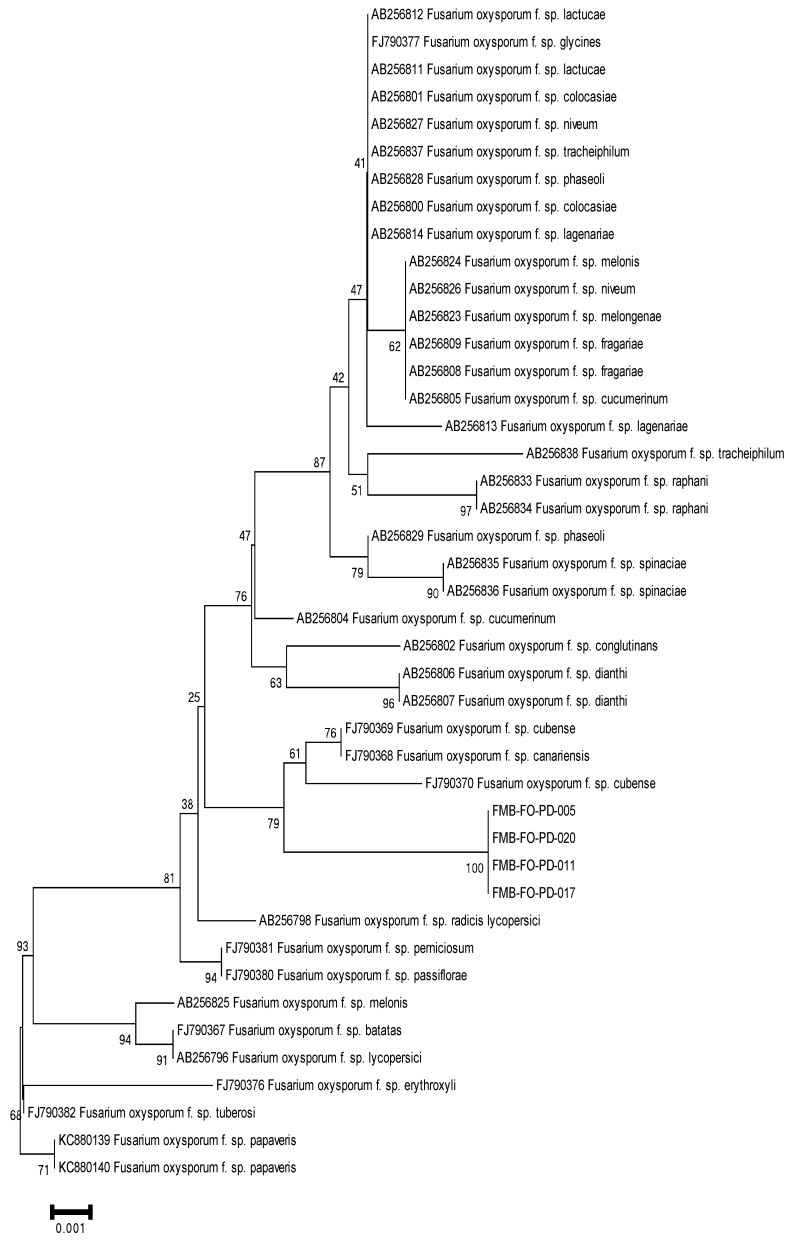
Phylogenetic tree based on the Polygalacturonase gene (Pgx4), using MEGA6.06 software under Neighbor-Joining (NJ) analysis, displayed a distinct and separate clade of the *F. oxysporum* isolates from other forma specialis with 100% bootstrap support.

**Figure 5:**
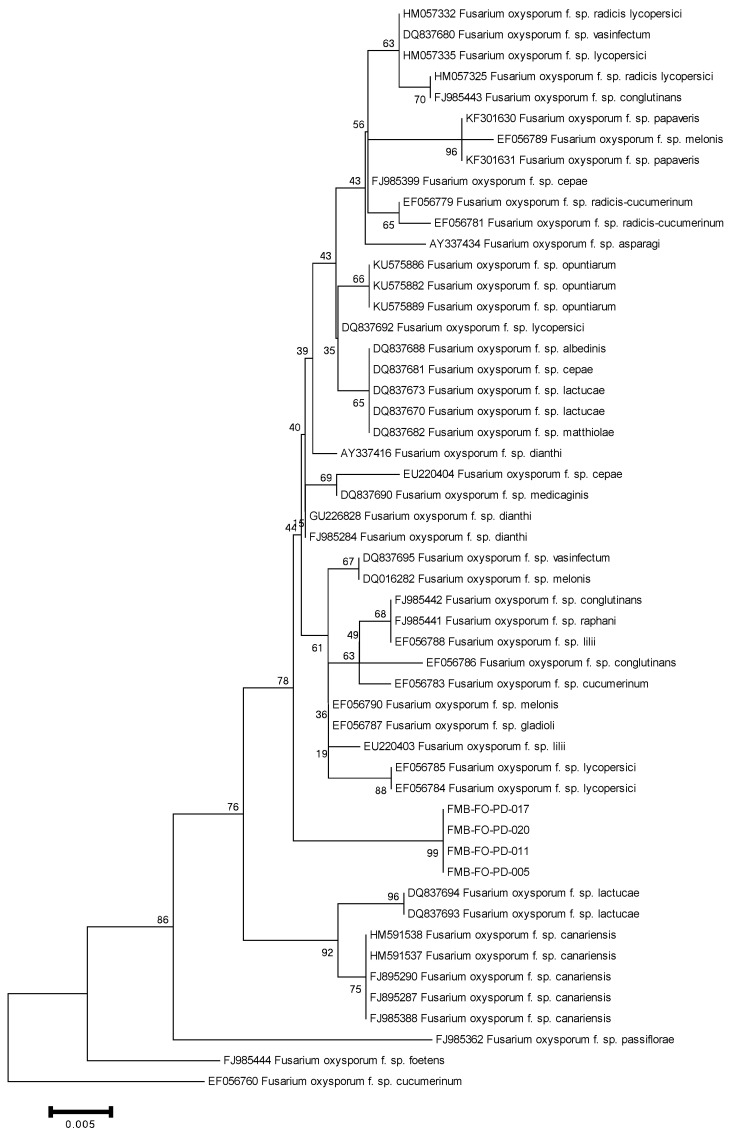
Phylogenetic tree based on translation elongation factor 1-α (TEF1-α) region, using MEGA6.06 software under Neighbor-Joining (NJ) analysis, displayed a distinct and separate clade of the *F. oxysporum* isolates from other forma specialis with 99% bootstrap support.

After this, these *F. oxysporum* isolates were delineated as forma specialis based on host pathogenicity. Forma specialis are morphologically indistinguishable, and their characterization at the subspecies level commonly revolved around bioassays; however, molecular approaches have made the taxonomic branches more authenticated. Polygalacturonases, TEF-1α, and IGS were used to distinguish the species at the subspecies level and corroborate the identification of a new forma specialis in *F. oxysporum* [20,21]. Hence, for delimiting the *F. oxysporum* (causes wilt in datepalm) taxonomy to the subspecies level, we analyzed endopolygalacturonase genes (Pg1, Pg5), exopolygalacturonase genes (Pgx1, Pgx4), translation elongation factor 1-α (TEF-1α), and intergenic spacer (IGS) region of rDNA in phylogenetic analysis. The PCR analysis amplified the DNA fragments of ~700 bp (TEF-1α), ~1500 bp (Pg1 and Pgx4), and ~2000 bp (IGS, Pg5, and Pgx1). We did not obtain the sequencing of Pg1, Pg5, and Pgx1, which might be due to sequencing errors. The sequencing errors lead to a low sequencing rate and/or no sequencing that could be due to high heterozygosity in genetic regions, homopolymeric sequences, runs of G or C in the regions, stops in the regions, and polymerase slippage during the Sanger sequencing method [26,27,28,29]. The sequences deposited in GenBank were assigned the accession numbers given in Supplementary Table S3. The phylogenetic trees constructed using MEGA6.06 under Neighbor-Joining (NJ) analysis revealed our fungal isolates in a well-separated clade from other *F. oxysporum* forma specialis. The phylogenetic trees based on IGS and Pgx4 genic regions delineated the *F. oxysporum* isolates in a separate clade from other forma specialis with 100% bootstrap support (Figure 3 and Figure 4). However, a phylogram based on TEF-1α separated these isolates with 99% bootstrap support from other members of the hierarchical tree (Figure 5). The *F. oxysporum* f. sp. albedinis documented for datepalm wilt has made cladding with *F. oxysporum* f. sp. cepae, *F. oxysporum* f. sp. lactucae, and *F. oxysporum* f. sp. matthiolae with 65% bootstrap support in a separate clade. The hierarchal analysis for identifying forma specialis has delineated our *F. oxysporum* isolates as strikingly distinct from *F. oxysporum* f. sp. albedinis and makes a distinct clade with 99% bootstrap support. 

The result of this study was congruent with the results of research conducted by Bertetti et al. [21], in which they used TEF1-α and IGS to identify new *F. oxysporum* forma specialis. Similarly, in another study by Ortu et al. [20], translation elongation factor 1-alpha and polygalacturonases genes were used to identify new types of *F. oxysporum* forma specialis. This study corroborates the authenticity of these genetic regions to delineate new types of forma specialis in *F. oxysporum*. The phylograms based on these genes revealed *F. oxysporum* isolates as a distinct new forma specialis in *F. oxysporum* (causes vascular wilt in datepalm) and registered as *Fusarium oxysporum* f. sp. *dactyliferum* in MycoBank with the accession number MycoBank #840871. 

### 3.2. Taxonomic Classification

*Fusarium oxysporum f. sp. dactyliferum* I.U Haq, S. Ijaz, I.A. Khan and N. A. Khan, f. sp. nov. MycoBank #840871.

*Material examined*: Pakistan, Punjab, from living fronds and roots of datepalm, 10 August 2019, I.U Haq (FMB H 12.1, Holotype, ex-type culture FMBCC 12.1= FMB-FO-PD-005, FMB-FO-PD-011, FMB-FO-PD-017, FMB-FO-PD-020).

Note: The forma specialis is preserved in a metabolically inactive state. Etymology: Named after the host (Phoenix dactylifera, botanical name) from which it was isolated.

## 4. Conclusions

The integrative pathogenicity postulate criteria will help where traditional pathogenicity tests cannot be completed. We have proven them with solid experimentation by exploring the plant and pathogen genome and transcriptome. We have tried to reshape Koch postulates’ features with omics’ help and fill the loopholes of Koch postulates that make put the pathogenicity in question due to systemic microbes. The highly specific nature of pathogenicity-related genes is exploited in these postulates and was proven and validated by probing the plant transcriptome, while the bipartite interaction between host and pathogen is present when the “gene for gene” concept keeps commands of the plant defense system during plant–pathogen interaction. Moreover, phylogenetic analysis of the authenticated pathogen proved that it is a new forma specialis of *F. oxysporum* that causes wilt and decline in datepalm.

## Data Availability

Not applicable.

## Supplementary Materials

The following supporting information can be downloaded at: https://www.mdpi.com/article/10.3390/plants11192643/s1, Table S1: Primer pairs used for amplification and quantitative detection of target sequences (genes). Table S2: A List of Fusarium isolates, and Fusarium species, used in this study for constructing phylograms. Table S3. A List of Fusarium oxysporum forma specialis, used in this study for constructing phylograms.
