# Supplementary material for: Integrative Pathogenicity Assay and Operational Taxonomy-Based Detection of New Forma Specialis of Fusarium oxysporum Causing Datepalm Wilt
Source: Plants (Basel). 2022 Oct 8;11(19):2643. doi: 10.3390/plants11192643 (PMC9571862; doi:10.3390/plants11192643)
Supplement: Supplementary file 1 [file plants-11-02643-s001.zip › Supplementary Table S1.pdf]

## Table S1: Primer pairs used for amplification and quantitative detection of target sequences (genes)

| Target | Primer Name | Forward primer sequence (5'→3') | Reverse primer sequence (5'→3') | Annealing Temperature | Reference |
|---|---|---|---|---|---|
| **PCR** | | | | | |
| ITS | ITS1-F/ITS4 | CTTGGTCATTTAGAGGAAGTAA | TCCTCCGCTTATTGATATGC | 54°C | White et al., 1990 |
| RPBII | fRPB2-cF/fRPB2-11aR | ATGGG(T/C)AA(A/G)CAAGC(T/C)ATGGG | GC(A/C)TGGATCTT(A/G)TC(A/G)TC(C/G)ACC | 54°C | Liu et al., 1999 |
| TEF-1α | Ef1/ Ef2 | ATGGGTAAGGAAGACAAGAC | GGAAGTACCAGTGATCATGTT | 53°C | O'Donnell et al., 1998 |
| IGS | CNS1/CNL12 | CCAGAGTGCCGATACCGATT | GCTTAGYGAACAKGGAGTG | 56°C | Appel and Gordon, 1995 |
| Pg1 | endoF/endoR2 | CCAGAGTGCCGATACCGATT | GCTTAGYGAACAKGGAGTG | 55°C | Hirano and Arie, 2009 |
| Pg5 | PG2F/PG2R | AGATGCAAGGCCGATGATGT | TCCATGTACTTCTCCTCACC | 55°C | Hirano and Arie, 2009 |
| Pgx1 | PgxF/PgxR | TCGTGGGGTAAAGCGTGGT | TTACTATAGGTCGATCAGCC | 57°C | Hirano and Arie, 2009 |
| Pgx4 | exoF2/exoR | TTACTGTCCACGAATGAGAAG | ACCCCAACCCCCCTCATCT | 55°C | Hirano and Arie, 2009 |
| SIX1 | F/R | ATGGTACTCCTTGGCGCCCTC | TGACAATGCGACCACGCCTCG | 54°C | Meldrum et al. 2012 |
| SIX2 | SIX2F2/ SIX2R2 | CAACGCCGTTTGAATAAGCA | TCTATCCGCTTTCTTCTCTC | 54°C | Lievens et al. 2009 |
| SIX3 | SIX3F1/ SIX3R2 | CCAGCCAGAAGGCCAGTTT | GGCAATTAACCACTCTGCC | 54°C | Lievens et al. 2009 |
| SIX4 | SIX4F1/ SIX4R1 | TCAGGCTTCACTTAGCATAC | GCCGACCGAAAAACCCTAA | 54°C | Lievens et al. 2009 |
| SIX5 | SIX5F1/ SIX5R1 | ACACGCTCTACTACTCTTCA | GAAAACCTCAACGCGGCAAA | 54°C | Lievens et al. 2009 |
| SIX6 | SIX6F1/ SIX6R1 | CTCTCCTGAACCATCAACTT | CAAGACCAGGTGTAGGCATT | 54°C | Lievens et al. 2009 |
| SIX7 | SIX7F1/ SIX7R1 | CATCTTTTCGCCGACTTGGT | CTTAGCACCCTTGAGTAACT | 54°C | Lievens et al. 2009 |
| SIX8 | F/R | TCGCCTGCATAACAGGTGCCG | TTGTGTAGAAACTGGACAGTCGATGC | 55°C | Meldrum et al. 2012 |
| SIX9 | F/R | CTTCTAGCAGTTGTAGCCAC | GTACGCCATTGACGCAAG | 54°C | Laurence et al. 2015 |
| SIX10 | F/R | AAAAAGCAGGCTCCATGAAGCTCTTGTGGTTG | AGAAAGCTGGGTCCTACTTAGACCTGGTAATTGTT | 58°C | Laurence et al. 2015 |
| SIX11 | F/R | GATGTTCTCCAAAGCCATCC | AGAATGCCACTCGGTGTGA | 54°C | Laurence et al. 2015 |
| SIX12 | F/R | CTAACGAAGTGAAAAGAAGTCCTC | GCCTCGCTGGCAAGTATTTGTT | 54°C | Taylor et al. 2016 |
| SIX13 | F/R | CCTTCATCATCGACAGTACAACG | ATCAAACCCGTAACTCAGCTCC | 54°C | Taylor et al. 2016 |
| SIX14 | F/R | TTGCCACCTATGCATACCG | TCCACATTCCTAAGCGAACC | 54°C | Laurence et al. 2015 |
| **RT-PCR** | | | | | |
| SIX1 | F/R | CCCTCTCAATCCTTGGGTTT | TGGGATGCTGCCACTTTATC | | In this study |
| SIX3 | F/R | GCTGATGCCGATTCATCTCT | GGTGCTGAAGCTCGTAGTAAA | | In this study |

| | | | | |
|---|---|---|---|---|
| SIX6 | F/R | GTGACCTTGTAGAGTGGAAGAC | CACAATGGTACCCAGATGATGA | In this study |
| SIX7 | F/R | CTTTAGGCCCAACGAAAGGA | TCCGGTGCCGATACCTAATA | In this study |
| SIX10 | F/R | AATTGCCGACTCTGGTGTATC | CTGTATGGCTCGTCTCGTTTAG | In this study |

| | |
|---|---|
| ITS | Internal Transcribed Spacer |
| TEF1-α | Translation Elongation Factor 1-alpha |
| RPBII | RNA polymerase II second largest subunit |
| IGS | Intergenic spacer |
| Pg1 | Endopolygalacturonase |
| Pg5 | Endopolygalacturonase |
| Pgx1 | Exopolygalacturonase |
| Pgx4 | Exopolygalacturonase |
| SIX | Secreted in xylem |
