# Supplementary material for: Integrative Pathogenicity Assay and Operational Taxonomy-Based Detection of New Forma Specialis of Fusarium oxysporum Causing Datepalm Wilt
Source: Plants (Basel). 2022 Oct 8;11(19):2643. doi: 10.3390/plants11192643 (PMC9571862; doi:10.3390/plants11192643)
Supplement: Supplementary file 1 [file plants-11-02643-s001.zip › Supplementary Table S2.pdf]

Supplementary Table S2: A **List of *Fusarium* isolates, and *Fusarium* species, used in this study for constructing phylograms**

Species/Isolate	Strain Accession #	GenBank Accession Numbers			Country
		ITS	TEF1-α	RPBII	
Isolate-02	FMB-FO-PD-005	OM319651.1	OP342612	OP234307	Sindh, Pakistan
Isolate-27	FMB-FO-PD-011	OM327774.1	OP342613	OP234308	Punjab Zone II, Pakistan
Isolate-30	FMB-FO-PD-017	OM391976.1	OP342614	OP234309	Punjab Zone I, Pakistan
Isolate-59	FMB-FO-PD-020	OM391977.1	OP342615	OP234310	KPK, Pakistan
Fusarium oxysporum	CBS132475	MH866023.1	KR071771.1	KU604313.1	South Africa
Fusarium proliferatum	CBS138981	KT716199.1	KT716210	KT716196.1	_
Fusarium proliferatum	CBS184.33	MH855401.1	KU604399.1	KU604244.1	Guyana
Fusarium proliferatum	CBS182.32	MH855271.1	KU604395.1	KU604246.1	USA
Fusarium zealandicum	CBS111.93	NR_138298.1	HQ728148.1	HM626684.1	_
Fusarium solani	CBS132898	KF255440	KF255484	KF255523	_
Fusarium falciforme	CBS135512	KM401895.1	KM401894.1	KM401892.1	_
Fusarium falciforme	CBS132191	KF255424.1	KF255467.1	KF255510.1	_
Fusarium solani	CBS118931	JX435204.1	JX435154.1	JX435254.1	_
Fusarium ambrosium	CBS571.94	KM231801.1	KM231929.1	KM232368.1	India

Fusarium neocosmosporiellum	CBS517.71	KM231804.1	KM231932.1	KM232371.1	India
Fusarium neocosmosporiellum	CBS325.54	KM231803.1	KM231931.1	KM232370.1	South Africa
Fusarium neocosmosporiellum	CBS562.70	KM231805.1	KM231933.1	KM232372.1	Guinea
Fusarium striatum	CBS101573	KM231798.1	KM231927.1	KM232365.1	New Zealand
Fusarium illudens	CBS119605	KM231806.1	KM231935.1	KM232374.1	New Zealand
Fusarium phaseoli	CBS265.50	KM232375.1	HE647964.1	KM232375.1	USA
Fusarium petroliphilum	CBS135955	KR071702.1	KU711768.1	KU604337.1	_
Fusarium globosum	CBS431.97	LT746280.1	LT746232.1	LT746345.1	South Africa
Fusarium globosum	CBS429.97	LT746278.1	LT746230.1	LT746343.1	South Africa
Fusarium fujikuroi	CBS119855	KR071669.1	KU711679.1	KU604260.1	_
Fusarium fujikuroi	CBS221.76	KR071666.1	KR071741.1	KU604255.1	Taiwan
Fusarium acutatum	CBS402.97	MH862652.1	KU604454.1	KT154005.1	_
Fusarium incarnatum	CBS132895	KF255437.1	KF255481.1	KF255546.1	_
Fusarium equiseti	CBS30794	MH862468.1	KR071777.1	KU604327.1	Germany

FMB= FMB-Fungal Molecular Biology Laboratory the Culture Collection University of Agriculture Faisalabad,

CBS=CBS-Culture Collection of the Centraalbureau voor Schimmelcultures, Fungal Biodiversity Centre, Utrecht, The Netherlands
