# Supplementary material for: Integrative Pathogenicity Assay and Operational Taxonomy-Based Detection of New Forma Specialis of Fusarium oxysporum Causing Datepalm Wilt
Source: Plants (Basel). 2022 Oct 8;11(19):2643. doi: 10.3390/plants11192643 (PMC9571862; doi:10.3390/plants11192643)
Supplement: Supplementary file 1 [file plants-11-02643-s001.zip › Supplementray Table S3.pdf]

Supplementary Table S3. A List of *Fusarium oxysporum* forma specialis, used in this study for constructing phylograms

Forma Specialis	Isolates/strain	Host	Country	GenBank Accession Number		
				TEF-1alpha	IGS	Pgx4
Fusarium oxysporum f.sp. albedinis	NRRL26622	Date palm		DQ837688	-	-
Fusarium oxysporum f.sp. papaveris	811/11IFR	Poppy	Italy	KF301630	-	KC880139
Fusarium oxysporum f.sp. papaveris	658/11IFR	Poppy	Italy	KF301631	-	KC880140
Fusarium oxysporum f. sp. opuntiarum	DB210211-26M	Insect	Italy	KU575889	-	-
Fusarium oxysporum f. sp. opuntiarum	DB210211-18M	Insect	Italy	KU575886	KU575872	-
Fusarium oxysporum f.sp. opuntiarum	DB14OTT05 M1	Insect	Italy	KU575882	-	-
Fusarium oxysporum f. sp. cepae	NRRL22538	Onion	USA	DQ837681	DQ831891	-
Fusarium oxysporum f.sp. cepae	NRRL 38481	Onion	USA	FJ985399	-	-
Fusarium oxysporum f.sp. lactucae	BMP1375	lettuce	USA	DQ837673	-	-
Fusarium oxysporum f. sp. lactucae	BMP1880	lettuce	USA	DQ837670	-	-
Fusarium oxysporum f.sp. lycopersici	FOLR2	Tomato	USA	DQ837692	DQ831894	-
Fusarium oxysporum f.sp. lycopersici	OSU451	Tomato	USA	HM057335	-	-
Fusarium oxysporum f. sp. asparagi	FOA50	Grass	Canada	AY337434	-	-
Fusarium oxysporum f.sp. melonis	CBS 423.90	Melon	Belgium	EF056789	-	-
Fusarium oxysporum f.sp. matthiolae	NRRL22545		USA	DQ837682	DQ831899	-
Fusarium oxysporum f.sp. radicis-cucumerinum	strain 30	Cucumber	Belgium	EF056781	-	-
Fusarium oxysporum f.sp. radicis-cucumerinum	strain 14	Cucumber	Belgium	EF056779	-	-
Fusarium oxysporum f. sp. vasinfectum	NRRL25231	Cotton	USA	DQ837680	DQ831896	-
Fusarium oxysporum f.sp. radicis lycopersici	CL-06202	Tomato	USA	HM057332	-	-
Fusarium oxysporum f.sp. radicis_lycopersici	CL-0620	Tomato	USA	HM057325	-	-
Fusarium oxysporum f.sp. conglutinans	NRRL 53158	Cabbage	USA	FJ985443	FJ985678	-
Fusarium oxysporum f.sp. dianthi	ATCC 64922	Carnation	Canada	AY337416	-	-
Fusarium oxysporum f.sp. cepae	NL106-2	Onion	Uruguay	EU220404	-	-
Fusarium oxysporum f.sp. medicaginis	NRRL22546	Alfalfa	USA	DQ837690	-	-
Fusarium oxysporum f.sp. vasinfectum	FOV14	Cotton	USA	DQ837695	-	-
Fusarium oxysporum f.sp. melonis	0348	Melon	USA	DQ016282	-	-
Fusarium oxysporum f.sp. melonis	CBS 420.90	Melon	Belgium	EF056790	-	-
Fusarium oxysporum f.sp. gladioli	NRRL 26993	Gladiolus	Belgium	EF056787	-	-
Fusarium oxysporum f.sp. lilii	Fol-11	Lily	Uruguay	EU220403	-	-

Fusarium oxysporum f.sp. lycopersici	MUCL 22544	Tomato	Belgium	EF056785	-	-
Fusarium oxysporum f.sp. lycopersici	MUCL 14159	Tomato	Belgium	EF056784	-	-
Fusarium oxysporum f.sp. cucumerinum	ATCC 16416	Cucumber	Belgium	EF056783	-	-
Fusarium oxysporum f.sp. conglutinans	strain 81-4	Cabbage	Belgium	EF056786	-	-
Fusarium oxysporum f.sp. lilii	NRRL 28395	Lily	Belgium	EF056788	-	-
Fusarium oxysporum f.sp. conglutinans	NRRL 53156	Cabbage	USA	FJ985442	-	-
Fusarium oxysporum f.sp. raphani	NRRL 53154	Radish	USA	FJ985441	-	-
Fusarium oxysporum f.sp. lactucae	FK09701	lettuce	USA	DQ837694	-	-
Fusarium oxysporum f.sp. lactucae	F9501	lettuce	USA	DQ837693	DQ831893	-
Fusarium oxysporum f.sp. canariensis	PLM-511A	Canary Island date palm	USA	HM591538	-	-
Fusarium oxysporum f._sp. canariensis	4873C	Canary Island date palm	USA	FJ895290	-	-
Fusarium oxysporum f.sp. canariensis	2675A	Canary Island date palm	USA	FJ895287	-	-
Fusarium oxysporum f.sp. canariensis	NRRL 38338	Canary Island date palm	USA	FJ985388	-	-
Fusarium oxysporum f.sp. foetens	NRRL 38302	ornamental plant	USA	FJ985444	-	-
Fusarium oxysporum f.sp. cucumerinum	strain 0016	Cucumber	Belgium	EF056760	-	-
Fusarium oxysporum f.sp. dianthi	NRRL 26222	Carnation	USA	FJ985284	-	-
Fusarium oxysporum f.sp. dianthi	C9-10	Carnation	Spain	GU226828	-	-
Fusarium oxysporum f.sp. callistephi	NRRL22536	Aster	USA	-	DQ831897	-
Fusarium oxysporum f.sp. callistephi	NRRL 22536	Aster	-	-	FJ985452	-

Fusarium oxysporum f.sp. narcissi	Fus113	Daffodil	USA	-	FJ981680	-
Fusarium oxysporum f.sp. matthiolae	FR-4A/02	Rocket	Italy	-	GU001827	-
Fusarium oxysporum f. gladioli	NRRL 26990	Gladiolus	USA	-	FJ985519	-
Fusarium oxysporum f. sp. fabae	FV-1/06	Lamb's lettuce	Italy	-	GQ914754	-
Fusarium oxysporum f.sp. radicis_lycopersici	NRRL 26775	Tomato	USA	-	HM057272	-
Fusarium oxysporum f.sp. lycopersici	BE1	Tomato	USA	-	HM057278	-
Fusarium oxysporum f.sp. gladioli	Fus074	Gladiolus	USA	-	FJ981676	-
Fusarium oxysporum f.sp. chrysanthemi	NRRL 36225	Chrysanthemum	USA	-	FJ985567	-
Fusarium oxysporum f.sp. vasinfectum	NRRL 38542	Cotton	USA	-	FJ985642	-
Fusarium oxysporum f.sp. vasinfectum	NRRL 32897	Cotton	USA	-	FJ985551	-
Fusarium oxysporum f.sp. fragariae	NRRL 26438	Strawberry	USA	-	FJ985503	-
Fusarium oxysporum f. sp. raphani	FR-0/02	Rocket	Italy	-	GU001824	-
-Fusarium oxysporum f.sp. raphani	Fus055	Radish	USA	-	FJ972807	-
Fusarium oxysporum f.sp. raphani	FR-33/03A	Rocket	Italy	-	GU001854	-
Fusarium oxysporum f.sp cucumerinum	NRRL 38591	Cucumber	USA	-	FJ985613	-
Fusarium oxysporum f.sp. cucumerinum	NRRL 26437	Cucumber	USA	-	FJ985502	-
Fusarium oxysporum f.sp. conglutinans	ATCC 52557	Cabbage	Italy	-	GQ914767	-
Fusarium oxysporum f.sp. conglutinans	ATCC 58385	Cabbage	Italy	-	GQ914770	-

Fusarium oxysporum f.sp. dianthi	NRRL 28365	Carnation	Italy	-	FJ985528	-
Fusarium oxysporum f.sp. basilici	NRRL 38318	Basil Seed	USA	-	FJ985615	-
Fusarium oxysporum f.sp. opuntiarum	31M	Insect	Italy	-	KU575868	-
Fusarium oxysporum f.sp. opuntiarum	CBS 743.79	Insect	Italy	-	KU575869	-
Fusarium oxysporum f.sp. opuntiarum	NRRL28934	Insect	USA	-	DQ831884	-
Fusarium oxysporum f.sp. opuntiarum	DB13GIU05-22M	Insect	Italy	-	KU575870	-
Fusarium oxysporum f.sp. opuntiarum	NRRL 28368	Insect	Italy	-	FJ985530	-
Fusarium oxysporum f. sp. lycopersici	MAFF103041	Tomato	Japan	-	-	-
Fusarium oxysporum f. sp. melonis	MAFF305544	Melon	Japan	-	-	AB256825
Fusarium oxysporum f. sp. tulipae	MAFF235109	Tulip	Japan	-	-	-
Fusarium oxysporum f. sp. tulipae	MAFF235105	Tulip	Japan	-	-	-
-Fusarium oxysporum f. sp. lycopersici	Saitama ly1	Tomato	Japan	-	-	-
Fusarium oxysporum f. sp. radicis lycopersici	KEF-2R1	Tomato	Japan	-	-	-
Fusarium oxysporum f. sp. dianthi	MAFF103072	Carnation	Japan	-	-	AB256806
Fusarium oxysporum f. sp. conglutinans	MAFF744001	Cabbage	Japan	-	-	-
Fusarium oxysporum f. sp. tracheiphilum	MAFF235726	Soyabean	Japan	-	-	AB256838
Fusarium oxysporum f. sp. raphani	MAFF103058	Radish	Japan	-	-	AB256833
Fusarium oxysporum f. sp. raphani	FR-1/05	Rocket	Italy	-	GU001860	-
Fusarium oxysporum f. sp. raphani	MAFF305124	Radish	Japan	-	-	AB256834

Fusarium oxysporum f. sp. lactucae	MAFF744028	Lettuce	Japan	-	-	AB256811
Fusarium oxysporum f. sp. melongenae	MAFF103051	Egg plant	Japan	-	-	AB256823
Fusarium oxysporum f. sp. melonis	MAFF305122	Melon	Japan	-	-	AB256824
Fusarium oxysporum f. sp. melongenae	MAFF103051	Egg plant	Japan	-	-	-
Fusarium oxysporum f. sp. fragariae	MAFF727510	strawberry	Japan	-	-	AB256809
Fusarium oxysporum f. sp. niveum	MAFF305543	Watermelon	Japan	-	-	AB256826
Fusarium oxysporum f. sp. tracheiphilum	MAFF235725	Soyabean	Japan	-	-	AB256837
Fusarium oxysporum f. sp. spinaciae	MAFF103059	Spinach	Japan	-	-	AB256835
Fusarium oxysporum f. sp. phaseoli	MAFF235727	Beans	Japan	-	-	AB256828
Fusarium oxysporum f. sp. niveum	NBRC9969	Watermelon	Japan	-	-	AB256827
Fusarium oxysporum f. sp. colocasiae	MAFF744032	Taro	Japan	-	-	AB256800
Fusarium oxysporum f. sp. phaseoli	NBRC9970	Beans	Japan	-	-	AB256829
Fusarium oxysporum f. sp. cucumerinum	MAFF103054	Cucumber	Japan	-	-	AB256804
Fusarium oxysporum f. sp. spinaciae	MAFF731044	Spinach	Japan	-	-	AB256836
Fusarium oxysporum f. sp. lagenariae	MAFF744002	Calabash gourd	Japan	-	-	AB256814
Fusarium oxysporum f. sp. radicis lycopersici	Saitama rly	Tomato	Japan	-	-	AB256798
Fusarium oxysporum f. sp. dianthi	MAFF305946	Carnation	Japan	-	-	AB256807
Fusarium oxysporum f. sp. colocasiae	MAFF744035	Taro	Japan	-	-	AB256801

Fusarium oxysporum f. sp. lagenariae	MAFF103008	Calabash gourd	Japan	-	-	AB256813
Fusarium oxysporum f. sp. lactucae	MAFF744029	Lettuce	Japan	-	-	AB256812
Fusarium oxysporum f. sp. fragariae	MAFF305557	Strawberry	Japan	-	-	AB256808
Fusarium oxysporum f. sp. cucumerinum	MAFF744005	Cucumber	Japan	-	-	AB256805
Fusarium oxysporum f. sp. conglutinans	MAFF727516	Cabbage	Japan	-	-	AB256802
Fusarium oxysporum f. sp. lycopersici	Saitama ly2	Tomato	Japan	-	-	AB256796
Fusarium oxysporum f. sp. glycines		Soybean	Belgium	-	-	FJ790377
Fusarium oxysporum f. sp. cubense	NRRL 25603	Banana	Belgium	-	-	FJ790369
Fusarium oxysporum f. sp. cubense	NRRL 26029	Banana	Belgium	-	-	FJ790370
Fusarium oxysporum f. cubense	NRRL 36107	Banana	-	FJ985326	-	-
Fusarium oxysporum f. sp. canariensis	NRRL 26035	Canary Island date palm	Belgium	-	-	FJ790368
Fusarium oxysporum f. sp. canariensis	PLM-385B	Canary Island date palm	USA	HM591537	-	-
Fusarium oxysporum f. sp. perniciosum	NRRL 22550	Mimosa	Belgium	-	-	FJ790381
Fusarium oxysporum f. sp. passiflorae	NRRL 22549	Passion Fruit	Belgium	-	-	FJ790380
Fusarium oxysporum f. sp. batatas	NRRL 26409	Sweet potato	Belgium	-	-	FJ790367
Fusarium oxysporum f. sp. tuberosi	NRRL 22555	Potato	Belgium	-	-	FJ790382
Fusarium oxysporum f. sp. erythroxyli	NRRL 26574	Coca	Belgium	-	-	FJ790376

Isolates	Specimen voucher	Host	Substrate	Country	GenBank Accession Number		
					TEF1-α	IGS	Pgx4
Isolate-02	FMB-FO-PD-005	Phoenix dactylifera (Date palm)	Fronds	Pakistan	OP342612	OP263620	OP293334
Isolate-27	FMB-FO-PD-011	Phoenix dactylifera (Date palm)	Roots	Pakistan	OP342613	OP263621	OP293335
Isolate-30	FMB-FO-PD-017	Phoenix dactylifera (Date palm)	Roots	Pakistan	OP342614	OP263622	OP293336
Isolate-59	FMB-FO-PD-020	Phoenix dactylifera (Date palm)	Fronds	Pakistan	OP342615	OP263623	OP293337
